# Primary Paratesticular Osteosarcoma: Case Report and A Review of the Literature

**DOI:** 10.1100/tsw.2007.155

**Published:** 2007-04-30

**Authors:** Anwar Al-Masri, Mubarak Al-Shraim, Ahmad Abu Al-Samen, Runjan Chetty, Andrew Evans

**Affiliations:** ^1^ Department of Pathology, King Hussein Medical Centre, The Royal Medical Services, Amman, Jordan; ^2^ Department of Laboratory Medicine and Pathobiology, University Health Network, Toronto, Ontario, Canada

**Keywords:** paratesticular osteosarcoma, metastasis

## Abstract

Primary paratesticular osteosarcoma is an extremely rare malignancy and, to date, only a few cases have been reported. To our knowledge, no such case arising directly from paratesticular soft tissue has been described. We describe a 52-year-old man who presented with left scrotal swelling and an excisional biopsy that revealed a sarcomatous lesion. Left inguinal orchiectomy was performed and histologic examination of the extensively sampled lesion revealed a pure paratesticular osteosarcoma. The patient died 6 months after the diagnosis with disseminated disease.

## INTRODUCTION

The paratesticular region contains a variety of anatomical structures like the spermatic cord, testicular tunics, epididymis, and Mullerian vestigial remnants, such as the appendices of the of epididymis and testis[1].

Paratesticular tumors can be clinically indistinguishable from testicular tumors, particularly when they are large. They usually present as a scrotal mass or swelling, with or without pain. In some cases, they can be misinterpreted clinically as an inguinal hernia. Most paratesticular tumors are benign (70%) and only about 30% are malignant. Although the exact site of origin of the tumor in the paratesticular region is often difficult to localize with certainty, it is thought that paratesticular sarcomas arise from the spermatic cord in 90% of cases[1]. Most paratesticular tumors are of mesenchymal origin[2] and can manifest as a variety of histologic types. Leiomyosarcomas, rhabdomyosarcomas, and liposarcomas are the most frequently described paratesticular sarcomas[3]. Rhabdomyosarcoma and embryonal sarcoma occur almost exclusively in children and young adults, while leiomyosarcoma, liposarcoma, and fibrosarcoma are more prevalent in the older adult population[4]. Primary extraskeletal osteosarcomas of the genitourinary tract are rare. Very few case reports describing primary osteosarcoma of the spermatic cord are to be found in the literature[4,5]. We report a case of primary paratesticular osteosarcoma in which the patient died 6 months after the diagnosis.

## CASE REPORT

A 52-year-old man presented to the surgical department with a painless, left scrotal mass that had been steadily enlarging over a 4-month period. The mass had extended to the left groin in the weeks prior to his presentation. There was no history of trauma or any medical illness of significance. Physical examination revealed a hard, nontender, and irregular left scrotal mass extending into the left inguinal area. The other testis was unremarkable. The clinical impression was of a chronic testicular hematoma. Whole-body imaging studies were unremarkable. The patient underwent a left inguinal orchiectomy, however, no retroperitoneal lymph nodes were dissected. A diagnosis of pure paratesticular osteosarcoma was made. Subsequent chemotherapy including a combination of cisplatin, doxorubicin, and methotrexate was given, however, the patient died after 6 months with extensive retroperitoneal lymph node metastases and inoperable intestinal obstruction.

## FINDINGS

### Gross Examination

The specimen consisted of a 17- × 10- × 10-cm hemorrhagic, nodular mass with an attached 7.0-cm testis and 8.0-cm length of spermatic cord. Serial sectioning revealed a well-circumscribed mass that was solid, firm, and gray-tan with extensive necrosis. The testis, epididymis, as well as the spermatic cord were unremarkable. In particular, there was no suggestion that the mass arose from any of these structures.

### Microscopic Examination

Twenty-five sections were submitted and revealed a highly vascular tumor with osteoid and bone formation (Figs. 1 and 2). There was extensive necrosis (Fig. 3). The tumor was extremely cellular with large, anaplastic, round-to-spindle cells with eosinophilic cytoplasm, vesicular nuclei, and prominent nucleoli. The tumor contained numerous multinucleated giant cells and high mitotic activity (Fig. 4). The testis, epididymis, and spermatic cord were histologically unremarkable. The features of the tumor were those of a pure paratesticular osteosarcoma. Vascular space invasion was present and all resection margins were free of tumor.

## DISCUSSION

Intrascrotal sarcomas are rare neoplasms, and have been traditionally divided into paratesticular and intratesticular tumors. Most testicular sarcomas arise from a teratoma or, less commonly, from a spermatocytic seminoma[6].

Paratesticular sarcomas are most commonly leiomyosarcomas (32%), rhabdomyosarcomas (24%), and liposarcomas (20%)[7,8]. Rhabdomyosarcomas are seen exclusively in the pediatric population[9,10]. Paratesticular sarcomas in adults are reported very infrequently and tend to occur in the elderly[10,11] where they are thought to arise from supportive stroma and paratesticular structures[11]. They are almost always unaccompanied by other components[6]. A few reports of paratesticular fibrosarcomas[10,12] and malignant fibrous histiocytomas[12] are present in the literature. To the best of our knowledge, only two reports of primary osteosarcomas of the spermatic cord are described in the literature[4,5].

Careful clinical and radiological evaluation is mandatory in such cases to exclude the possibility of a primary osseous source for the tumor. In our case, extensive clinical and radiological workup did not reveal a skeletal lesion. The prognosis of paratesticular sarcomas is variable from case to case with reports describing early metastasis[10] and multiple recurrences[13], as well as long-term disease-free survival[10].

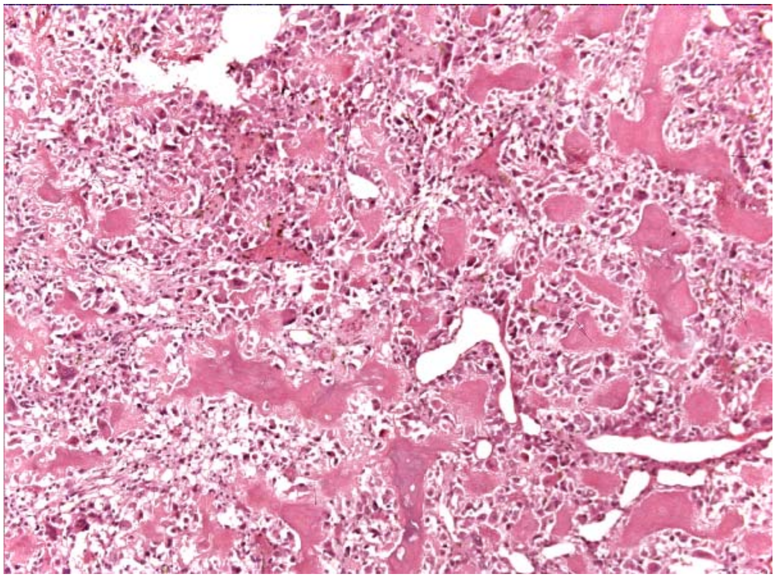

FIGURE 1

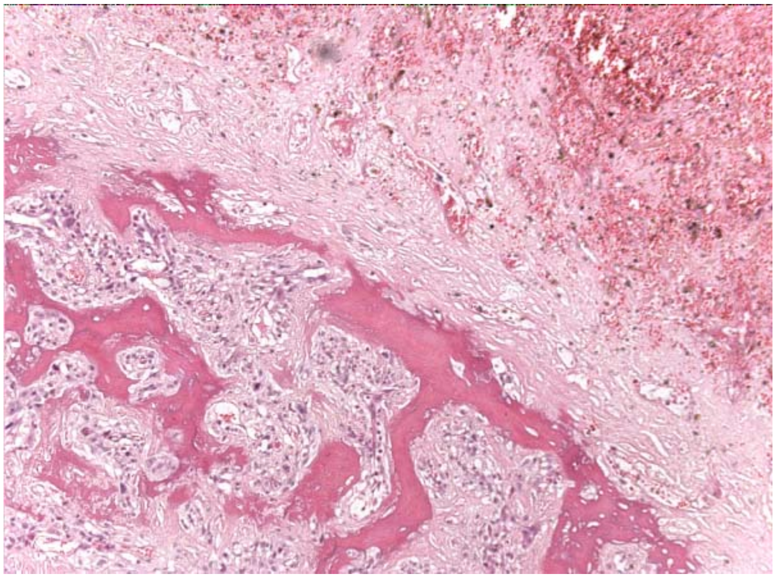

FIGURE 2

**FIGURES 1 and 2.** Photomicrograph H&E (×100 original magnification) showing the osteoid and bone formation.

Adjuvant chemotherapy is clearly indicated in the treatment of patients with skeletal osteosarcoma[14]. The local and systemic treatment of paratesticular sarcomas remains controversial, but the general consensus for the management of such tumors in adults is complete resection with high ligation of the spermatic cord[1]. In the described cases of primary spermatic cord osteosarcomas[4], the patients did not receive adjuvant systemic chemotherapy and followed uneventful clinical courses following wide surgical resection. Although the literature for the surgical modalities to treat these tumors is limited, a retroperitoneal lymph node dissection (RPLND) was not performed in our case, as the general health condition of the patient was too poor to allow such surgical approach.

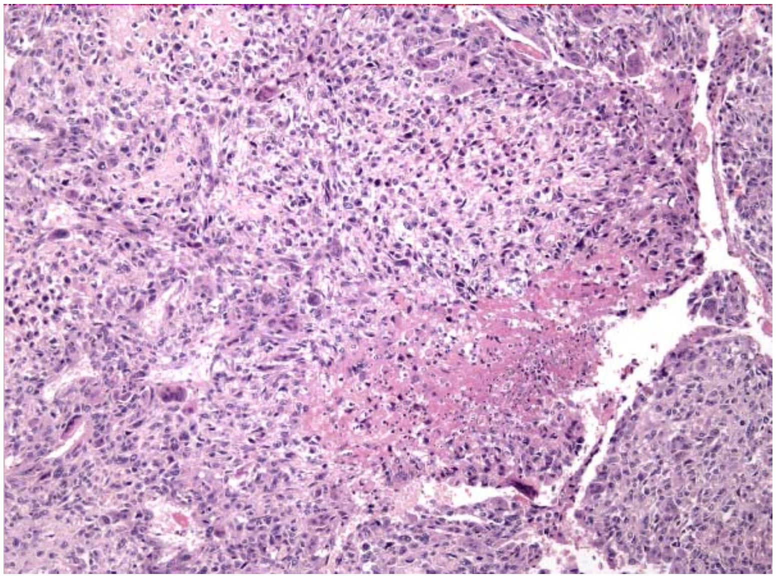

**FIGURE 3.** Photomicrograph H&E (×100 original magnification) showing extensive necrosis.

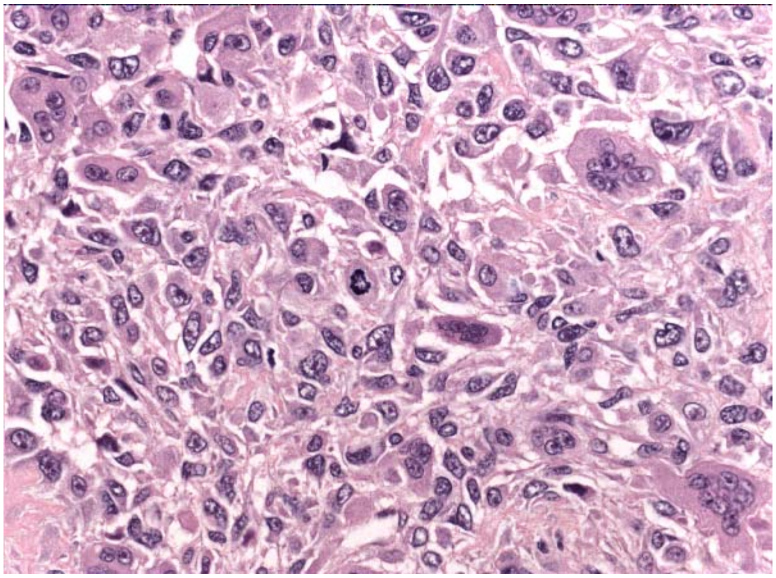

**FIGURE 4**. Photomicrograph H&E (×400 original magnification) showing the osteoclast type giant cells and mitotic activity.

Intratesticular sarcomas are similarly rare and are usually associated with a germ cell tumor[6], where they likely arise as part of a sarcomatous transformation of a gonadal germ cell tumor[15,16,17]. They may arise *de novo* or postchemotherapy[15,17,18], and the specific entities include rhabdomyosarcomas[6], leiomyosarcomas[6], angiosarcomas[6,19], and undifferentiated sarcomas[6]. Intratesticular sarcomas that are not associated with a germ cell tumor are very rare[6] and have been described in an older age group[6]. Most of these have been rhabdomyosarcomas[20,21,22], leiomyosarcomas[22,23], spindle cell sarcoma[22], fibrosarcoma[22], chondrosarcoma[22], and osteosarcomas[22,24]. They appear to have a better overall prognosis[6]. Pure testicular sarcoma should only be diagnosed after extensive sampling to exclude a coexisting germ cell tumor component[6].

## CONCLUSION

Primary pure paratesticular osteosarcoma is an extremely rare, stromal malignancy. It appears to follow an aggressive course with potential for distant metastases and is associated with a poor prognosis.
